# Helios expressing regulatory T cells are correlated with decreased IL-2 producing CD8 T cells and antibody diversity in Mozambican individuals living chronically with HIV-1

**DOI:** 10.1186/s12865-022-00487-3

**Published:** 2022-03-14

**Authors:** Raquel Matavele Chissumba, Cacildo Magul, Rosa Macamo, Vânia Monteiro, Maria Enosse, Ivalda Macicame, Victória Cumbane, Nilesh Bhatt, Edna Viegas, Michelle Imbach, Leigh Anne Eller, Christina S. Polyak, Luc Kestens, for the RV363 Study Group, Ilesh Jani, Julie Ake, Khelvon De Araujo, Nilesh Bhatt, Igor Capitine, Raquel Matavele Chissumba, Alberto Machaze, Eduardo Namalamgo, Celso Castiano, Emelva Manhiça, Mirna Mutombene, Ducília Matimbe, Onélia Guiliche, Vania Mapossa, Vania Monteiro, Nilzio Cavele, Trevor Crowell, Leigh Anne Eller, Zebiba Hassen, Michelle Imbach, Luis Inhambizo, Qun Li, Ivalda Macicame, Ferrao Mandlate, Carmélia Massingue, Mark Milazzo, Vanessa Monteiro, Chiaka Nwoga, Christina S. Polyak, Patrícia Ramgi, Merlin L. Robb, Steve Schech, Gail Smith, Edith M. Swann, Edna Viegas, Adam Yates

**Affiliations:** 1 Instituto Nacional de Saúde, Distrito de Marracuene, Estrada Nacional N°1, Marracuene, Província de Maputo, Mozambique; 2 Institute of Tropical Medicine, Department of Biomedical Sciences, Antwerp, Belgium; 3 Department of Biomedical Sciences, University of Antwerp, Antwerp, Belgium; 4 U.S. Military HIV Research Program, Walter Reed Army Institute of Research, Silver Spring, MD USA; 5 Henry M. Jackson Foundation for the Advancement of Military Medicine, Bethesda, MD USA

**Keywords:** Seronegativity, Tregs, HIV chronic infection, Helios

## Abstract

**Background:**

Human immunodeficiency virus type 1 (HIV-1) causes impairment of T and B cell responses, which begins during the acute phase of infection and is not completely restored by antiretroviral treatment. Regulatory T cell (Tregs) can improve overall disease outcome by controlling chronic inflammation but may also suppress beneficial HIV-1 specific immune responses. We aimed to analyze the profile of Tregs and their correlation with the status of T cells activation, the expression of IL-2 and IFNγ and the profile of HIV-1 specific antibodies response in Mozambican people living chronically with HIV-1 (PLWH-C).

**Results:**

In PLWH-C, the proportion of total Tregs was positively correlated with the proportion of IL-2^+^CD4 T cells (r = 0.647; *p* = 0.032) and IL-2^+^IFNγ^+^CD8 T cells (r = 0.551; *p* = 0.014), while the proportions of Helios^+^Tregs correlated inversely with levels of IL-2^+^CD8 T cells (r = − 0.541; *p* = 0.017). Overall, PLWH-C, with (82%) or without virologic suppression (64%), were seronegative for at least HIV-1 p31, gp160 or p24, and the breadth of antibody responses was positively correlated with proportions of CD38^+^HLA-DR^+^CD8 T cells (r = 0.620; *p* = 0.012), viral load (r = 0.452; *p* = 0.040) and inversely with absolute CD4 T cells count (r = − 0.481; *p* = 0.027). Analysis of all individuals living HIV-1 showed that the breadth of HIV-1 antibody responses was inversely correlated with the proportion of Helios^+^Tregs (r = − 0.45; *p* = 0.02).

**Conclusion:**

Among Mozambican people living with HIV-1, seronegativity to some HIV-1 proteins is common, particularly in virologically suppressed individuals. Furthermore, lower diversity of HIV-specific antibodies is correlated to lower immune activation, lower viral replication and higher CD4 counts, in PLWH-C. Elevation in the proportion of Helios^+^Tregs is related to a reduction of CD8 T expressing intracellular IL-2, in PLWH-C, but may contribute to impairment of B cell function.

**Supplementary Information:**

The online version contains supplementary material available at 10.1186/s12865-022-00487-3.

## Introduction

Human Immunodeficiency Virus type 1 (HIV-1) infection causes generalized immunodeficiency which is characterized by a profound depletion of CD4 T cells, impairment of B and T cells function, and systemic immune activation which persists during the chronic phase of the disease [1, 2]. During the acute phase of HIV-1 infection, disruption of the gut mucosa, associated with depletion of CD4 T cells at the gut epithelium, is proposed to result in massive microbial translocation into the blood [3, 4]. This culminates with activation of innate and adaptive mediators of the immune response, and a persistent inflammatory environment and immune exhaustion [5, 6].

Chronic immune activation induced by HIV-1 infection also alters the lymphoid tissue architecture leading to impairment of immune reconstitution [5]. Additionally, there is a loss of HIV-specific memory B cells, probably associated with loss of HIV-specific CD4 T cells, which is not restored by antiretroviral therapy (ART) [7, 8]. It has also been postulated that effective virologic control with ART may lead to loss of anti-HIV antibodies as a consequence of reduced antigenic stimulation [9]. However, a report of eighty-four people living chronically with HIV-1 (PLWH-C) and undetectable viral loads for longer than five years under ART showed that seroreversion measured using HIV-1 Western blot is a very rare event occurring in only one patient [10]. Thus, the significance of reduced HIV-1 specific antibody reactivity on clinical diagnostic assays, particularly in PLWH-C remains to be determined.

Regulatory T cells (Tregs) are a subset of CD4 T cells with the potential to suppress T and B cell functions by a vast array of mechanisms such as IL-2 deprivation and secretion of suppressor mediators [11]. Tregs are identified by expression of various markers including transcription factor Foxp3 and the α chain of IL-2 receptor (CD25) [11]. Other markers, including Helios and CD45RA are associated with Tregs function [12–14]. Expression of the transcription factor Helios identifies stable and highly suppressive Tregs [15, 16]. Deletion of Helios on Tregs in mice leads to progressive systemic immune activation, enhanced germinal center formation and conversion to effector T cells [15, 17]. We previously reported that in Mozambican people living early with HIV-1 (PLWH-E), at elevated proportion of Helios expressing Tregs, lower levels of HIV-1 viral replication and recovery of CD4 T cells absolute counts were observed [18]. This study aimed to assess how the profile of Tregs correlates with the profile of T cells and the breadth of circulating HIV-1 specific antibodies in PLWH-C.

## Results

### Study participant characteristics

An overview of the study participants is shown in Table 1. Eight PLWH-C without viral suppression (VS) were not receiving ART at the time of PBMC collection (median HIV-1 viral load was log_10_ 4.4 copies/mL (4.1–4.9)). Two PLWH-C on ART and with viral load of less than 20 viral RNA copies/mL were included in the group of individuals with VS. The absolute number of CD4 T cells was lower in PLWH-C with or without VS compared to people living without HIV-1 (PLWOH) study group.

**Table 1:** Characteristics of study participant groups

	PLWH-C without VS	PLWH-C with VS	PLWH-E	PLWOH
N	12	12	7	9
Age (years), median (IQR)	23 (21–28)	22 (18–27)	22 (21–30)	20 (19–24)
Sex (Female/Male)	7/5	5/7	4/3	5/4
#CD4 (cells/μL), Median (IQR)	477 (324–640)*	525 (462–628)*	604 (401–763)	763 (740–1265)
HIV-1 viral load (median log_10_ (IQR))	4.4 (4.1–4.9)	Undetectable or < 1.3	4.4 (3.1–5.6)	N/A
Time after first positive result** (months),	> 12	> 12	3	N/A
**# **Participants on ART (N)	4	12	0	N/A

*IQR* interquartile range. *M/F* male/female. *N/A* not applicable. *ART* antiretroviral therapy. *VS* viral suppression

^*^p < 0.05 compared to HIV negative group

^**^ HIV-1 diagnosis antibodies rapid test

We also measured the breadth of HIV-1 antibody response here described also as the number of HIV-1 antigens recognized by antibodies, at the analysis point. We observed seronegativity to the tested HIV-1 proteins in PLWH-C as shown in Table 2. All participants reacted to gp41. Overall, 28/29 (97%) participants reacted to gp160, 14/29 (48%) reacted to p31 and 19/29 (66%) reacted to p24. The proportion of individuals with absence of reactivity to at least one protein was 81.8% for PLWH-C with VS but 63.6% for PLWH-C without VS.

**Table 2:** Summary of the HIV-1 specific antibody profiles to four HIV-1 proteins in individuals living with HIV-1

	PLWH-C without VS	PLWH-C with VS		PLWH-E	Total
N	11	11		7	29
Reactivity to HIV-1 p31 (%)	63.6%	27.3%		28.0%	48.3%
Reactivity to HIV-1 gp160 (%)	100%	90.9%		100%	96.6%
Reactivity to HIV-1 p24 (%)	72.7%	45.5%		85.7%	65.5%
Reactivity to HIV-1 gp41 (%)	100%	100%		100%	100%
Non-reactive at least to one of the tested proteins (%)	63.6%	81.8%		57%	69%

### In individuals living with HIV-1 without virologic control, the proportions of CD8 T cells expressing IFNγ but not the activation markers HLA-DR and CD38, correlates better with viral loads

We evaluated co-expression of activation markers CD38 and HLA-DR in unstimulated CD4 and CD8 T cells and intracellular expression of IL-2 and IFNγ after stimulation with SEAB. Overall, the level of co-expression of HLA-DR and CD38 on CD4 and CD8 T cells was higher in PLWH-C compared to PLWOH (*p* = 0.005 and *p* = 0.019, respectively). However, after stratification in two groups, based on viral suppression status, we observed that this difference remained significant only in PLWH-C without VS for both activated CD4 and CD8 T cells (*p* = 0.003 and *p* < 0.001, respectively). As described in previous studies [19, 20] the frequency of co-expression of activation markers on CD8 T cells correlated with HIV**-**1 viral load (r = 0.711; *p* < 0.001).

**Fig. 1:**
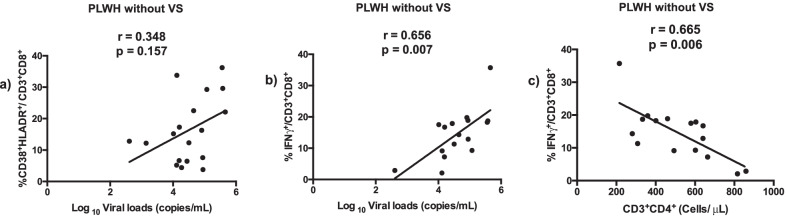
Correlation between activated T cells and viral loads in PLWH. PBMC from PLWH (18) without viral suppression, PLWH-C (11) and PLWH-E (7). PBMC were stained with monoclonal antibodies and analyzed by eight-color flow cytometer. Viral loads were determined by a clinically validated in vitro nucleic acid amplification test for the quantitation of HIV-1 RNA in human plasma as described in Methods section (**a**) Correlation between the viral loads and proportion of CD8 T cells co-expressing CD38 and HLA-DR in PLWH without VS. Correlation between the proportion of CD8 T cells expressing IFNγ with viral loads (**b**) and CD4 T cell counts (**c**) in PLWH without VS. Spearman’s correlation r and p-values are indicated (***p < 0.0001)

When evaluating all participants living with HIV without VS, including PLWH-E, the correlation between the frequency of co-expression of activation markers on CD8 T cells with HIV**-**1 viral loads was not observed (r = 0.348, *p* = 0.157) (Fig. 1a). However, we found a correlation between CD8 T cells expressing intracellular IFNγ with viral loads (r = 0.656, *p* = 0.007) and CD4 T cell counts (r = 0.665, *p* = 0.006) (Fig. 1b and 1c).

### Individuals living chronically with HIV-1, the breadth of HIV-1 antibody responses correlates with co-expression of activation markers on CD8 T cells, viral replication and absolute CD4 T cell counts

**Fig. 2:**
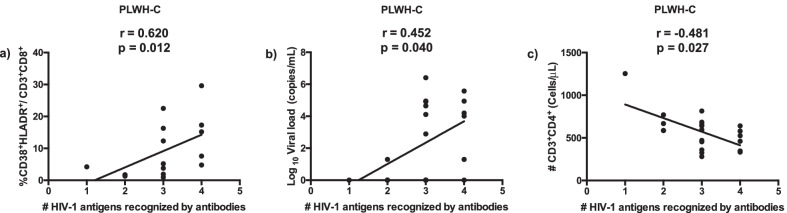
Correlations between the number of HIV-1 antigens recognized by antibodies and immune restoration indicators. PBMC and whole blood from PLWH-C (n = 20) were stained with monoclonal antibodies and analyzed by polychromatic flow cytometry. Serum antibody reactivity to HIV-1 – Env (gp160 and gp41), Gag (p24) and Pol (p31) proteins was analyzed on stored serum samples from all individuals living with HIV-1, using immuno-chromatographic test as described in Methods section. Correlations between the number (#) of HIV-1 antigens recognized by antibodies/ seroreactivity to HIV-1 proteins and (**a**) activation of CD8 T cells, (**b**) viral load and (**c**) absolute CD4 T cell counts in PLWH-C. Each point corresponds to one individual. Spearman’s correlation r and p-values are indicated on each figure

In PLWH-E, the number of HIV-1 antigens recognized by antibodies correlated inversely with activation of CD8 T cells and positively with CD4 T cell absolute counts [18]. Here we found that for our participants with chronic infection, the breadth of antibody response to HIV-1 proteins correlated positively with the levels of co-expression of HLA-DR and CD38 on CD8 T cells (r = 0.620; *p* = 0.012), HIV-1 viral load (r = 0.452; *p* = 0.040) and inversely with CD4 T cell counts (r = − 0.481; *p* = 0.027) (Fig. 2a–c). Co-expression of HLA-DR and CD38 on CD4 T cells did not correlate with the breadth of antibody response to HIV-1 proteins (r = 0.269; *p* = 0.328).

### The frequency of Helios expressing Tregs, but not total Tregs, correlate with decreased proportions of CD8 T cells producing IL-2 in people living chronically with HIV and decreased number of HIV-1 antigens recognized by antibodies

**Fig. 3:**
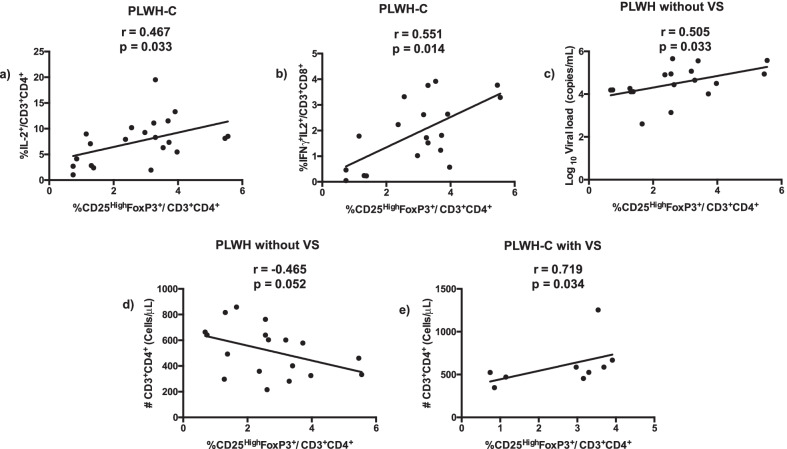
Correlations between Tregs and cytokine-producing CD4 and CD8 T cells, CD4 T cell absolute counts and viral loads. PBMC from PLWH-C were isolated and treated as described in Methods section. PBMC were stained with monoclonal antibodies and analyzed by polychromatic flow cytometry. Absolute counts of CD4 T cells were determined using a four-color flow cytometer as described in the methods section. Correlation between the proportion of Tregs and **a** frequency of CD4 T cells expressing IL-2 (n = 21) PLWH-C and **b** frequency of CD8 T cell expressing IL-2 combined with IFNγ (n = 19) PLWH-C. **c** Correlation between the proportions of Tregs within CD4 T cells and viral load (n = 18) and **d** CD4 T cells absolute counts in PLWH without VS (n = 18). **e** Correlation between the proportions of Tregs and CD4 T cells absolute counts in PLWH-C with VS (n = 9)

Persistent inflammation is considered the one of main promoters of immunologic failure in PLWH [5]. We measured the proportions of Tregs in unstimulated cells and the proportions of effector CD4 and CD8 T cells expressing IL-2 and IFNγ after stimulation of with SEAB. The proportions of Tregs in PLWH-C did not differ significantly from those observed in PLWOH (*p* = 0.213). The Tregs proportions in PLWH-C was correlated with the proportion of CD4 T cells expressing intracellular IL-2 (r = 0.467; *p* = 0.033) or CD8 T cells expressing IL-2 in combination with IFNγ (r = 0.551; *p* = 0.014) (Fig. 3a and b). When assessing all study participants living with HIV-1 but without VS, including PLWH-E, the same correlation between the proportions of Tregs and CD8 T cells, producing IL-2 combined with IFNγ was also observed (r = 0.574; *p* = 0.031). Furthermore, Tregs proportions were also correlated with proportions of CD8 T cells, producing IL-2 (r = 0.515, *p* = 0.043) and IFNγ (r = 0.512, *p* = 0.045) alone and with CD4 T cells expressing activation markers (r = 0.549, *p* = 0.018).

When evaluating all study participants living with HIV-1 but without VS, including PLWH-E we found a positive correlation between Tregs with the viral loads (r = 0.505, *p* = 0.033) and trended to correlate inversely with absolute CD4 counts (r = − 0.465; *p* = 0.052) (Fig. 3c and d). However, in PLWH-C but with VS, we found a positive correlation between Tregs and absolute CD4 T cells counts (r = 0.719, *p* = 0.034) (Fig. 3e).

**Fig. 4:**
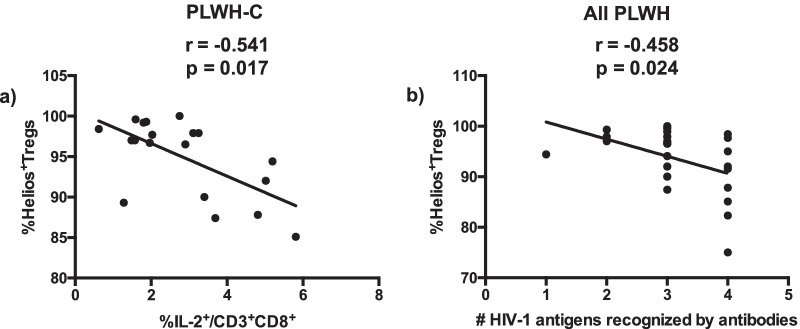
Correlation between proportions of Helios expressing Tregs (Helios^+^Tregs) and cytokine-producing CD8 T cells and seroreactivity to HIV-1 proteins. PBMC from PLWH were isolated and treated as described in the methods section. PBMC were stained with monoclonal antibodies and analyzed by polychromatic flow cytometry. The number of HIV-1 antigens, Env (gp160 and gp41), Gag (p24) and Pol (p31), recognized by antibodies, was analyzed on stored serum samples from all individuals living with HIV-1, using immuno-chromatographic test as described in Methods section. Viral loads were determined using plasma samples as described in the *Methods* section. Correlations between: **a** relative frequency of Helios expressing Tregs in PLWH-C and the proportion of CD8 T cells expressing IL-2 (n = 19). **b** proportions of Helios expressing Tregs and the number (#) of HIV-1 antigens recognized by antibodies for all PLWH (n = 24). Each data point corresponds to a single individual. Spearman’s correlation r and p-values are indicated on each figure

Regarding the Tregs expressing the transcription factor Helios we found an inverse correlation with the proportions of CD8 T cells expressing IL-2 (r = − 0.541; *p* = 0.017) (Fig. 4a). Moreover, when grouping all individuals living with HIV-1, including PLWH-E, we found that the proportion of Tregs expressing Helios correlated inversely with the breadth of antibodies response to HIV-1 proteins (r = − 0.458; *p* = 0.024) (Fig. 4b).

## Discussion

Early studies of HIV-specific CD8 T cells response in individuals living with HIV-1 showed an inverse correlation between the early emergence of CD8-specific responses and plasmatic viral levels [21]. However, the analysis of total HIV-, Env- and Nef-specific CD8 T cells producing IFNγ showed a positive correlation with viral load [22]. Further studies suggested that CD8 T cell response, specifically Gag-specific response, may have different roles according to the stage of the infection [23]. In this study, we also measured the levels of intracellular cytokines IL-2 and IFNγ in CD8 T cells stimulated with SEAB and found that the proportions of those expressing IFNγ correlated directly with HIV-1 viral load and inversely with absolute CD4 counts. Our results suggest that levels of IFNγ expression on CD8 T cells reflects T-cell activation driven by HIV-1 viral replication.

As observed in a previous report of early HIV infection [18] the proportion and absolute count of Tregs remained comparable in our cohort of PLWH-C relative to PLWOH, despite the lower frequencies and absolute counts of total CD4 T cells in PLWH-C. This could mean that during HIV-1 infection, Tregs might be preserved within the total CD4 T cells compartment. Indeed, studies have reported expansion of Tregs within CD4 T cells in people living with HIV-1, probably as a consequence of increased immune activation [24]. Furthermore, the unaltered proportion of Tregs could also be due to increased migration of Tregs from other tissues to the peripheral blood, as suggested by our previous results of increased expression of CXCR3 and CCR5 on Tregs [18], or a combination of factors.

Contrary to what we observed in people living with HIV without VS, including PLWH-E, the relative frequency of Tregs, in PLWH-C and with suppressed viral replication, correlated with higher absolute CD4 T cell counts. This observation raises the hypothesis that in conditions of VS, Tregs expand in parallel with the recovery of CD4 T cell counts. However, in spite of this apparent immune restoration in PLWH-C and suppressed viral replication, it seems that Tregs are not able to control immune activation, since higher levels of CD8 T cell producing pro-inflammatory cytokines are observed despite high levels of total Tregs in these individuals.

After observing that high frequency of IFNγ expression in CD8 T cells in people living with HIV-1 without VS, correlated with increased viral load, decreased CD4 counts and total Tregs, we assessed how the profile of these CD8 T cells correlated with expression of the transcription factor Helios in Tregs, which represents Tregs with a stable suppressive function. Although we did not find a significant correlation when evaluating IFNγ producing CD8 T cells, we found that in individuals with increased frequencies of Tregs expressing the transcription factor Helios, lower proportions of IL-2 expressing CD8 T cells were observed. These observations suggest that in chronic HIV-1 infection, Tregs expressing Helios may have the potential to suppress systemic inflammation. Suppression of activated or pro-inflammatory CD8 T cells could be one of the contributions of Helios^+^ Tregs controlling the deleterious effects of HIV-1 infection. This is aligned with the inverse correlation that we previously observed between Helios^+^ Tregs and plasmatic viral loads [18].

Here, we also found that decreased breadth of reactivity to HIV-1 proteins is a frequent event in subjects living with chronic HIV-1, more evident in those on ART, and is correlated with immune regulation and virologic control. During HIV infection, Tregs are thought to be important in limiting the levels of systemic immune activation, but Tregs can also suppress the development of HIV-specific protective T and B cell immune responses [24, 25]. When characterizing the antibody profile against different HIV-1 antigens, we observed that irrespective of infection for more than one year, participants living chronically with HIV-1 tended to show incomplete HIV-1 specific antibody profiles. This was more pronounced in virologically suppressed ART-treated individuals living chronically with HIV, with 80% presenting seronegativity to at least one of the tested HIV-1 proteins. However, a previous study reported seroreversion of HIV-1 antibodies as a rare event in PLWH-C [10]. These contradictions might be in part due to the assays and HIV target proteins used for each study. In that study the authors targeted all HIV-1 proteins, with remarks to gp160, gp41, p24 and gp120 and used Western blot assay while in our study we only targeted gp160, gp41, p24 and p31 and used a recombinant protein assay. Seroreversion of antibodies targeting HIV-1 proteins has been described in patients living with HIV-1 treated shortly after primary infection [9] [26–28]. However, our study was cross-sectional, and time of ART initiation was unknown so seroreversion could not be assessed. Seroreversion on some HIV-1 antibodies assays has also been reported in late stage disease when CD4^+^ T cell counts are extremely low [9, 29]. The cause of HIV-1 antibody loss has been attributed to reduced antigenic stimulation in individuals with effective virologic control by ART [9]. In this regard, we observed that the decrease in number of HIV-1 antigens recognized by antibodies, correlated with virologic control, decreased expression of activation markers on CD8 T cells and recovery of CD4 T cell counts. Thus, our observations raise the hypothesis that in chronic infection, while increased production of IL-2 and IFNγ is associated with laboratory indicators of disease progression, seronegativity to some HIV-1 proteins is associated with indicators of disease recovery.

With regard to the impact of Tregs on decreased seroreactivity to HIV-1, it has been suggested that accumulation of Tregs that secrete TGF-β1 in the lymph nodes attempting to control persistent inflammation are the major contributors of collagen deposition that characterizes chronic infection [1]. Collagen deposition in lymph nodes is associated with loss of follicular dendritic cells and fibroblastic reticular cells during the course of HIV-1/SIV infection [1]. We found that individuals with decreased seroreactivity to HIV-1 proteins had higher proportions of Tregs expressing Helios. We hypothesize that the relative frequency of Tregs expressing Helios, despite contributing to control of chronic inflammation, contribute to impaired B cell function and the ensuing seronegativity.

Altogether, our study results suggest that decreased seroreactivity to HIV-1 is frequent in ART-treated virologically suppressed individuals living chronically with HIV-1 and could be a reflection of decreased stimulation of HIV-1 specific B due to absence of viral antigen(s). Furthermore, our results raise the hypothesis that elevation of Tregs with higher suppressive potential, but not total Tregs, may have a beneficial impact controlling the levels of inflammation and viral loads but may compromise B-cell function. Further studies are needed to sustain that hypothesis.

The main limitations of this study are: (1) the small sample size and lack of same data for some participants due to failures during the experiments (2) the lack of information regarding the ART regimen, and the time from initiation of ART, (3) the duration of HIV-1 infection in participants living chronically with HIV-1 and the profile of reactivity to HIV-1 proteins at diagnosis, (4) assessment of a limited number of inflammatory markers, (5) lack of assessment of anti-HIV-1 T cell immune response and (6) the lack of assessment of Tregs subsets function.

## Methods

### Study participants

Forty participants enrolled in RV363 were included in this study. RV363 was a prospective study that assessed HIV-1 incidence, retention rate and willingness of adults to participate in future HIV vaccine trials in Mozambique, as previously described [30]. People living without HIV-1 (PLWOH), classified as high risk for HIV-1 acquisition, were screened every 3 months for HIV-1 antibodies over a 2-years period. For this study, participants were classified as PLWH-E, within 3 months after the first HIV-1 positive result and were classified as PLWH-C, those living with HIV for more than 12 months. Some of PLWH-C had detectable viral load whereas the other had viral suppression (VS) for at least one year. PLWOH were included in the study as control group. All study participants provided written informed consent to participate in this study.

### HIV diagnosis, profile of reactivity to HIV-1 proteins, CD4 counts and HIV-1 viral load testing

HIV-1 diagnosis was performed on fresh venous whole blood samples following the Mozambican national algorithm for HIV testing, which consists of two sequential rapid immunochromatographic tests for detection of anti-HIV-1/2 antibodies. The screening was first performed using the *Alere Determine™ HIV-1/2* (Abbott, USA) rapid test. Participants that the specimens did not react to the test were diagnosed as non-living with HIV-1. Reactive specimens were confirmed by a second rapid test (*Uni-Gold*® *HIV*, Trinity Biotech PLC, Ireland). Discordant results were resolved by a fourth-generation ELISA (*Genscreen Ultra HIV Ag-Ab* (BioRad, France) kit, using stored plasma or serum. The antibody reactivity pattern for HIV-1—Env (gp160 and gp41), Gag (p24) and Pol (p31) proteins was analyzed on stored serum samples from all individuals living with HIV-1, using the *Geenius HIV 1/2 Confirmatory Assay* (BioRad, France). CD4 T cells were enumerated from fresh EDTA-whole blood by four-color flow cytometry (FACS Calibur, Becton Dickinson [BD], USA). Plasma HIV-1 viral load was measured using the *COBAS AmpliPrep/COBAS TaqMan HIV-1 Test, v2* (Roche, USA).

### Peripheral blood mononuclear cells isolation

Peripheral blood mononuclear cells (PBMC) were isolated within 8 h of phlebotomy from heparin anti-coagulated blood using Ficoll-Paque Plus (GE Healthcare, Sweden) and Leucosep tubes (Greiner Bio-One, German). PBMC were cryopreserved in 10% dimethyl sulfoxide (DMSO) + 90% fetal calf serum (FCS) in liquid nitrogen at or below − 140 °C.

### In vitro stimulation of peripheral blood mononuclear cells

PBMC were thawed in a water bath at 37 °C and washed twice in complete RPMI medium supplemented with 20% FCS followed by 10% FCS. Viable cells were counted using a Nucleocounter (Chemometec, Denmark). PBMC were rested overnight in complete RPMI medium supplemented with 10% FCS (R10 medium) at 37 °C/ 7.5% CO2. Subsequently, 500,000 PBMC, suspended in 100 µL of R10 medium, were mixed with monensin, brefeldin A, and co-stimulatory monoclonal antibodies against CD28 (1 µg/mL) and CD49d (1 µg/mL) diluted in R10 medium. PBMC were then stimulated with 50 µL of Staphylococcus enterotoxin A and B (SEAB, (1 µg/mL)) or were maintained with 0.5% of DMSO in R10 medium (negative control). Cell suspensions were incubated for six hours at 37 °C/7.5% CO2 and subsequently stored overnight at 2–8 °C prior to analysis. Unstimulated PBMC were used for determination of T cells activation markers.

### Immunophenotyping

Following overnight exposure to antigen or medium, PBMC were treated with 20 mM EDTA and incubated for 15 min in the dark at room temperature. Cells were then washed with phosphate buffered saline (PBS) prior to staining with 50 μL of viability dye (fixable viability stain (FVS) 510, BD, USA). PBMC’s were kept in the dark at room temperature, for another 15 min, then washed twice with 5% FCS in PBS. After washing, PBMC were stained with monoclonal antibodies cocktails for identification of cell surface markers. Cells used for intracellular staining of cytokines and FoxP3 were fixed with 200 µL of 1X Human FoxP3 Buffer A (BD, USA) for 10 min at 4 °C. PBMC were then washed with diluted BD Perm/Wash buffer (BD, USA) and permeabilized with Human FoxP3 Buffer C 1× for 30 min in the dark at room temperature. Prior to staining of PBMC, for detection of intracellular marker, cells were washed twice with BD Perm/Wash buffer (BD, USA). The combinations of monoclonal antibodies for intracellular staining were added to the cells and incubated at 2–8 °C. After 30 min, cells were washed twice with Perm/Wash buffer (BD, USA) and finally treated with 200 µL of 1× BD CellFix (BD, USA). The following combinations of monoclonal antibodies and the viability dye, all from BD, USA, were used: (1) Activation Markers: CD3^FITC^/HLA-DR^PE−Cy7^/CD38^APC^/CD8^APC−H7^/CD4^V450^/FVS510; (2) Tregs: CD4^FITC^/FoxP3^PE^/CD45RA^PerCP−Cy5.5^/CD25^PE−Cy7^/Helios^APC^/CD3^APC−H7^/IFNγ^V450^/FVS510 and (3) Intracellular cytokine staining: CD3^FITC^/IFNγ^PE−Cy7^/IL2^APC^/CD8^APC−H7^/CD4^V450^/FVS510.

After 30 min, samples were analyzed on a FACSCanto II (BD, USA). A minimum of 100,000 events were acquired using Diva software version 8 (BD, USA). For quality control purposes, the BD Cytometer Setup & Tracking (CST) beads and BD Comp beads were used to ensure consistency of results over time and for compensation, respectively. The post-acquisition analyses, including compensation, were performed using FlowJo software, version 10 (FlowJo LLC, USA). Gating strategy for definition of Tregs is shown as Additional file 1: Fig. S1.

### Statistical analysis

Statistical analyses were performed using *GraphPad Prism* version 6.0 h (USA). The Mann–Whitney test was used to test heterogeneity among different groups. Correlations between two variables were performed by the Spearman Rank correlation. Differences or correlations with *p* values less than 0.05 were considered statistically significant.

## Supplementary Information

**Additional file 1**. **Supplementary figure 1:** Gating strategy for definition of Tregs by FlowJo. Gating strategy for: (a) identification of lymphocytes. (b) identification of singlets from lymphocytes. (c) definition of live cells from singlets. (d) identification of T cells from live cells. (e) identification of CD4 T cells from T cells. (f) definition of the region for positivity of Tregs based on FMO control for FoxP3 in total CD4 T cells. (g) identification of Tregs as CD25HighFoxP3+ from total CD4 T cell. (h) definition of Helios positive cells from Tregs.

## Data Availability

The datasets used and analyzed during the current study are available from the corresponding author on reasonable request.

## Abbreviations

ART: Antiretroviral therapy
CNBS: Mozambican National Committee on Bioethics in Health (Comité Nacional de Biotética em Saúde de Moçambique)
DMSO: Dimethyl sulfoxide
FCS: Fetal calf serum
FVS 510: Fixable viability stain 510
Foxp3: Transcription factor forkhead box P3
HIV-1: Human Immunodeficiency Virus type 1
PBMC: Peripheral blood mononuclear cells
PBS: Phosphate buffered saline
Tregs: Regulatory CD4^+^ T cells
PLWH-C: People living chronically with HIV-1
PLWH-E: People living early with HIV-1
PLWOH: People living without HIV-1
VS: Virologic suppression
