# Supplementary material for: Helios expressing regulatory T cells are correlated with decreased IL-2 producing CD8 T cells and antibody diversity in Mozambican individuals living chronically with HIV-1
Source: BMC Immunol. 2022 Mar 14;23:12. doi: 10.1186/s12865-022-00487-3 (PMC8922818; doi:10.1186/s12865-022-00487-3)
Supplement: Supplementary file 1 — Additional file 1. Supplementary figure 1: Gating strategy for definition of Tregs by FlowJo. Gating strategy for: (a) identification of lymphocytes. (b) identification of singlets from lymphocytes. (c) definition of live cells from singlets. (d) identification of T cells from live cells. (e) identification of CD4 T cells from T cells. (f) definition of the region for positivity of Tregs based on FMO control for FoxP3 in total CD4 T cells. (g) identification of Tregs as CD25HighFoxP3+ from total CD4 T cell. (h) definition of Helios positive cells from Tregs. [file 12865_2022_487_MOESM1_ESM.pptx]

## Slide 1
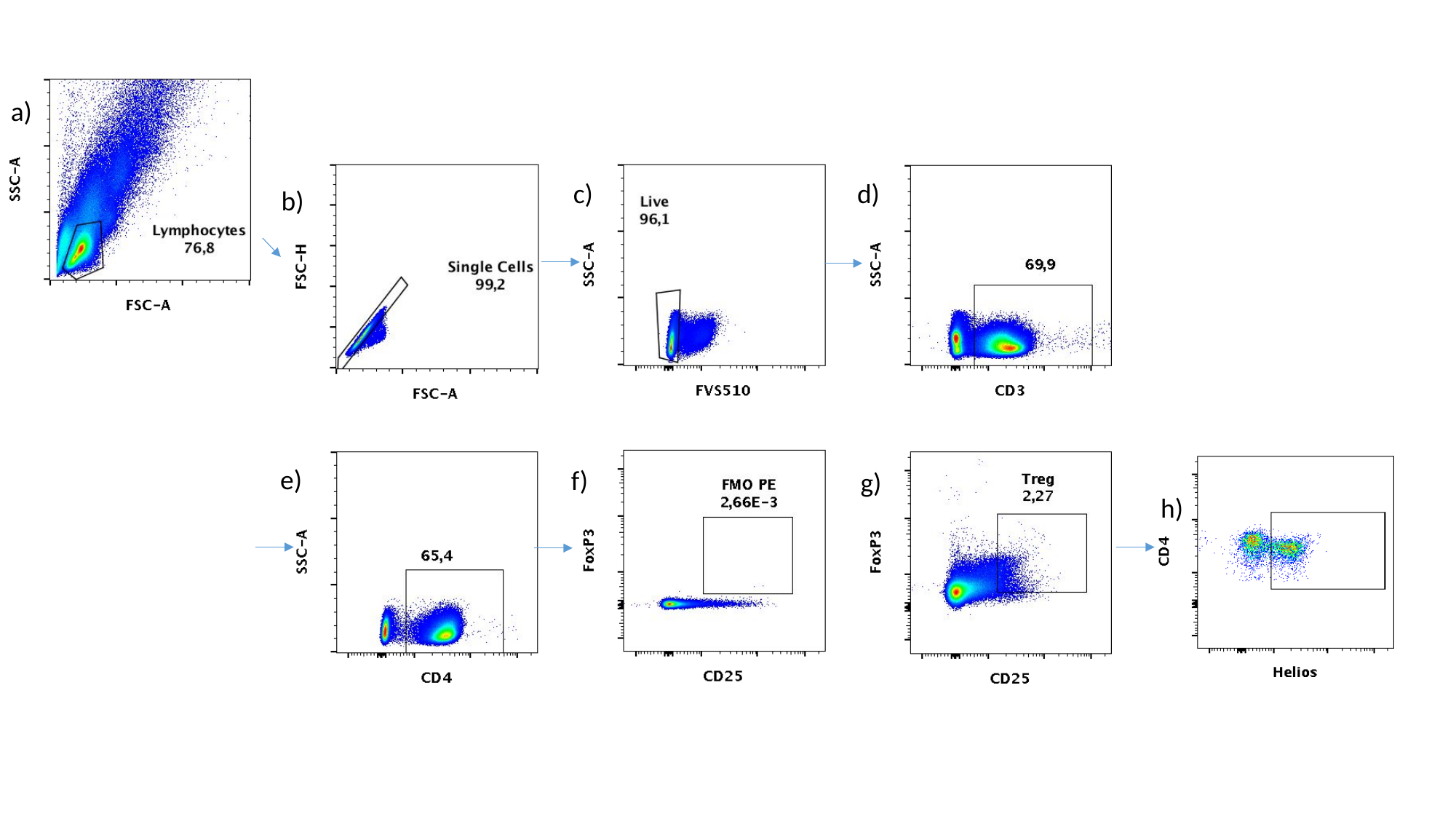

a)
c)
d)
b)
e)
f)
g)
h)

## Slide 2
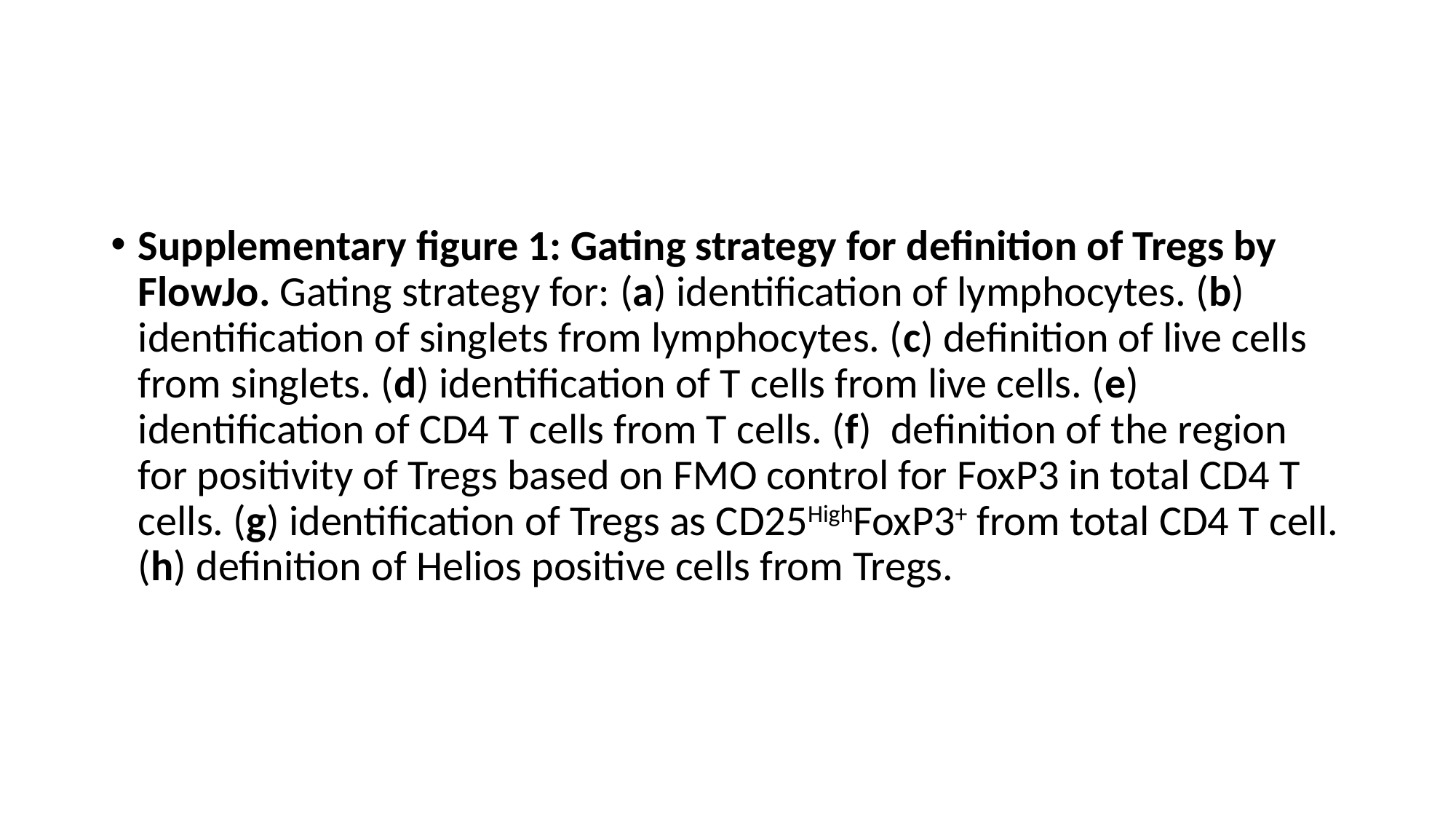

Supplementary figure 1: Gating strategy for definition of Tregs by FlowJo. Gating strategy for: (a) identification of lymphocytes. (b) identification of singlets from lymphocytes. (c) definition of live cells from singlets. (d) identification of T cells from live cells. (e) identification of CD4 T cells from T cells. (f) definition of the region for positivity of Tregs based on FMO control for FoxP3 in total CD4 T cells. (g) identification of Tregs as CD25HighFoxP3+ from total CD4 T cell. (h) definition of Helios positive cells from Tregs.
